# Formalin-evoked pain triggers sex-specific behavior and spinal immune response

**DOI:** 10.1038/s41598-023-36245-7

**Published:** 2023-06-12

**Authors:** Lucie Pepino, Pascale Malapert, Andrew J. Saurin, Aziz Moqrich, Ana Reynders

**Affiliations:** CNRS, Institut de Biologie du Développement de Marseille, UMR 7288, Case 907, Aix-Marseille Université, 13288 Marseille Cedex 09, France

**Keywords:** Cellular neuroscience, Sensory processing, Pain

## Abstract

Mounting evidence shows sex-related differences in the experience of pain with women suffering more from chronic pain than men. Yet, our understanding of the biological basis underlying those differences remains incomplete. Using an adapted model of formalin-induced chemical/inflammatory pain, we report here that in contrast to male mice, females distinctly display two types of nocifensive responses to formalin, distinguishable by the duration of the interphase. Females in proestrus and in metestrus exhibited respectively a short-lasting and a long-lasting interphase, underscoring the influence of the estrus cycle on the duration of the interphase, rather than the transcriptional content of the dorsal horn of the spinal cord (DHSC). Additionally, deep RNA-sequencing of DHSC showed that formalin-evoked pain was accompanied by a male-preponderant enrichment in genes associated with the immune modulation of pain, revealing an unanticipated contribution of neutrophils. Taking advantage of the male-enriched transcript encoding the neutrophil associated protein Lipocalin 2 (*Lcn2*) and using flow cytometry, we confirmed that formalin triggered the recruitment of LCN2-expressing neutrophils in the pia mater of spinal meninges, preferentially in males. Our data consolidate the contribution of female estrus cycle to pain perception and provide evidence supporting a sex-specific immune regulation of formalin-evoked pain.

## Introduction

Men and women experience pain differently. Women exhibit increased sensitivity to several acute pain modalities^1–3^ and are over-represented in various chronic pain conditions such as migraine, fibromyalgia, temporomandibular joint disorder and irritable bowel disorder^2–4^. Despite a well-established literature reporting quantitative sex differences in pain, the underlying biological mechanisms are not fully understood. Sex hormones play an important contribution, but more recent data put forward that other biological substrates involved in pain detection, transmission and modulation, including genes, neurons and immune cells, differently operate in males and females^5–7^. In rodents, quantitative sex differences have been documented in various pre-clinical model of pain, including in the formalin paradigm of inflammatory/chemical pain^8–13^. Intra-plantar injection of formalin elicits a spontaneous phasic nocifensive behavior (licking, flinching, biting of the paw) initiated by a short-lasting intense pain (first phase), followed by a time-lapse with reduced pain (interphase) and finally by a last phase during which pain resumes (second phase)^14^. The first phase is considered to represent the activation of nociceptive fibers, the interphase, the recruitment of endogenous pain-inhibitory mechanisms and the second phase, the spinal sensitization of the pain pathway^14–16^. Previous studies, mostly performed in rats, have shown that females exhibit increased formalin-induced pain^10,11,17^ and that males and females process differently formalin-evoked pain^13,18–20^. This is widely attributed to sex hormones, as shown by the absence of sex differences in gonadectomized subjects^11,17–19^. Importantly, in all of these studies, the same volume of 1 to 5% formalin is administrated to both sexes, and given that there are considerable weight differences between males and females of the same age (https://www.arc.wa.gov.au/?page_id=125), one may put forward that the initial intensity of the stimulus (formalin) is readily higher in females as compared to males. Considering this, in the present study, we adapted the volume of injected formalin to the weight of the mice, as previously described^21^, to determine sex differences in formalin-evoked pain behavior and to investigate the associated transcriptional modifications in the dorsal horn of the spinal cord (DHSC). Despite no sex differences in the amplitude of formalin-evoked pain, we report here that in contrast to males, females distinctly displayed two types of nocifensive responses, distinguishable by the duration of the interphase. This was mainly influenced by female estrus cycle, rather than by the transcriptional content of the DHSC. Notably, we found that proestrus and metestrus females exhibited respectively a short- and a long-lasting interphase. In addition, deep RNA-sequencing of DHSC tissues of formalin-exposed mice of each sex, showed a male preponderant enrichment of genes associated with the immune response, revealing an unanticipated contribution of neutrophils. Taking advantage of the male-enriched transcript encoding the neutrophil associated protein Lipocalin 2 (*Lcn2*) and using flow cytometry, we confirmed that formalin triggered the recruitment of LCN2-expressing neutrophils in the pia mater of spinal meninges, preferentially in males. Altogether, our data consolidate the contribution of female estrus cycle to pain perception and provide evidence supporting a sex-specific immune regulation of formalin-evoked pain.

## Results

### Female mice display two types of formalin-induced pain behaviors, respectively marked by short- and long-lasting interphase

**Figure 1:**
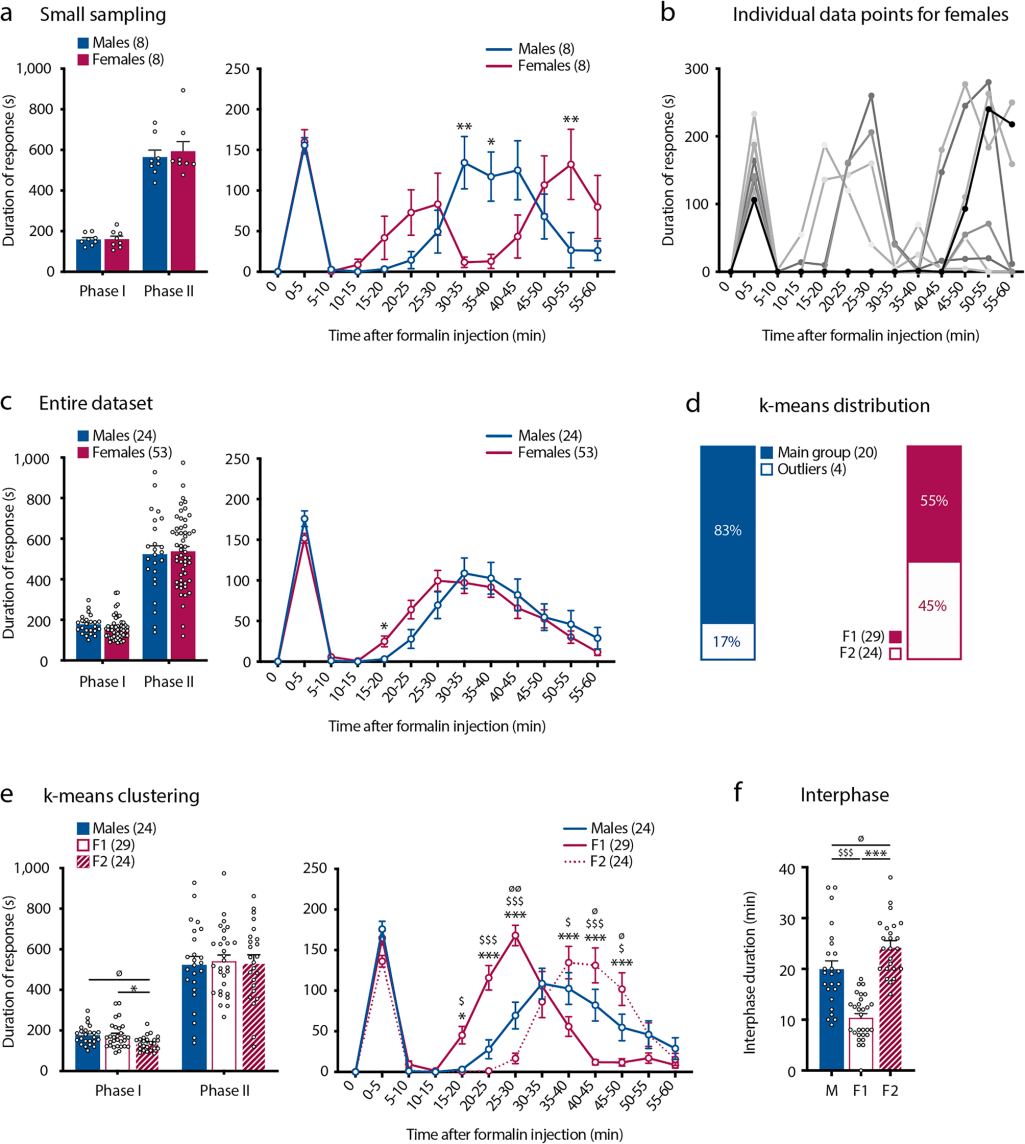
Female mice display two types of formalin-induced pain behaviors, respectively marked by short- and long-lasting interphases. (**a**) Cumulative duration (left) and the duration per 5 min intervals (time-course, right) of formalin-evoked pain behavior (in seconds, s) in a small cohort of n = 8 male and n = 8 female mice. Data are presented as mean ± SEM. (*p < 0.05; **p < 0.01). (**b**) Individual data for the time-course of formalin-evoked pain in the females included the (**a**). (**c**) Cumulative duration (left) and time-course (right) of formalin-evoked nocifensive behavior (in seconds, s) in a large group of mice (n = 24 males and 53 females). Data are presented as mean ± SEM. (*p < 0.05). (**d**) K-means based subdivision of males and females; shows the percentage of subjects in each sub-group (males and outlier males and F1 and F2 females). (**e**) Cumulative duration (left) and time-course (right) of formalin-evoked nocifensive behavior (in seconds, s) in F1 (n = 29) and F2 (n = 24) females and in males (n = 24). Data are presented as mean ± SEM. (M-F1: ^$^p < 0.05; ^$$$^p < 0.001) (M-F2: ^ø^p < 0.05; ^øø^p < 0.01) (F1-F2: *p < 0.05; ***p < 0.001). (**f**) Duration of the interphase in males (n = 24), F1 (n = 29) and F2 (n = 24) females (in minutes, min). Data are presented as mean ± SEM. (M-F1: ^$$$^p < 0.001) (M-F2: ^ø^p < 0.05) (F1-F2: ***p < 0.001).

The amplitude and time-course of formalin-evoked pain behavior was analyzed in female and male C57Bl/6 mice, using an adapted experimental setting, as previously described^21^. Each mouse received an intraplantar injection of a 2% formalin solution at a rate of 8 µl/20 g of weight and their pain behavior was monitored per 5 min intervals for 60 min. We started this study by analyzing relatively small cohorts of male and free cycling females (n = 8, per sex). Under these conditions, the total duration of formalin-evoked nocifensive behavior was equal between sexes (Fig. 1a). However, we observed differences in the time-course of pain responses (Fig. 1a). In males, formalin triggered a classical biphasic response with a short-lasting first phase, followed by an interphase and then a second phase (Fig. 1a). In contrast females exhibited three peaks of formalin-evoked nocifensive behavior, either corresponding to a tri-phasic response or underlining variability in the appearance of the second phase, reflecting a variability in the duration of the interphase (Fig. 1a). Analysis of individual responses clearly revealed two typical formalin-triggered nocifensive behaviors amongst females, with one group of females (hereafter called F1) exhibiting a short-lasting interphase and a second group (hereafter called F2), displaying a much longer-lasting interphase (Fig. 1b). In contrast, no subgroups were observable in males (https://figshare.com/articles/figure/K-means_clustering_for_males/22036304). We defined the interphase as the total duration of the quiescent period following the first phase and before the emergence of the second phase of the formalin-induced pain. Because large-enough datasets are required to increase the power of statistical analysis, especially when considering free-cycling females, we considerably increased the number of mice per sex (n = 24 males and 53 females). In this large sampling, formalin triggered a similar biphasic response in both sexes with no difference in the total duration of nocifensive behavior (Fig. 1c). However, when we analyzed the behavioral responses during the 5 min intervals, female mice started their second phase earlier than males (Fig. 1c). Despite this apparent homogeneity in the time-course of formalin-evoked pain response within females, the above observed typical F1 and F2 behaviors remained identifiable, as revealed by unbiased k-means clustering (Fig. 1d,e). Using this approach, we found that in 29 of the 53 females included in our study (55%), the second phase of formalin-evoked pain resumed after about 10 min and thus corresponded to the short-lasting interphase F1 group, while in the remaining 24 (45%) the second phase started at about 23 min post-formalin injection, consistent with what observed in the long-lasting interphase F2 group (Fig. 1e,f). We also noticed slight differences in the first phase of the formalin group, with F2 females exhibiting less formalin-evoked pain than F1 females (Fig. 1e). When applied to males, k-means clustering highlighted the outliers, as among the 24 male subjects, 20 of them (83%) exhibited similar formalin-evoked pain behavior (Fig. 1d). Of note, the mean duration of the interphase in males was 20 min (Fig. 1f). Together, these data demonstrate sex-related differences in the behavioral responses to formalin-induced pain.

### Estrus cycle influences formalin-evoked pain behavior in females, with minimal impact on gene expression levels in the DHSC

**Figure 2:**
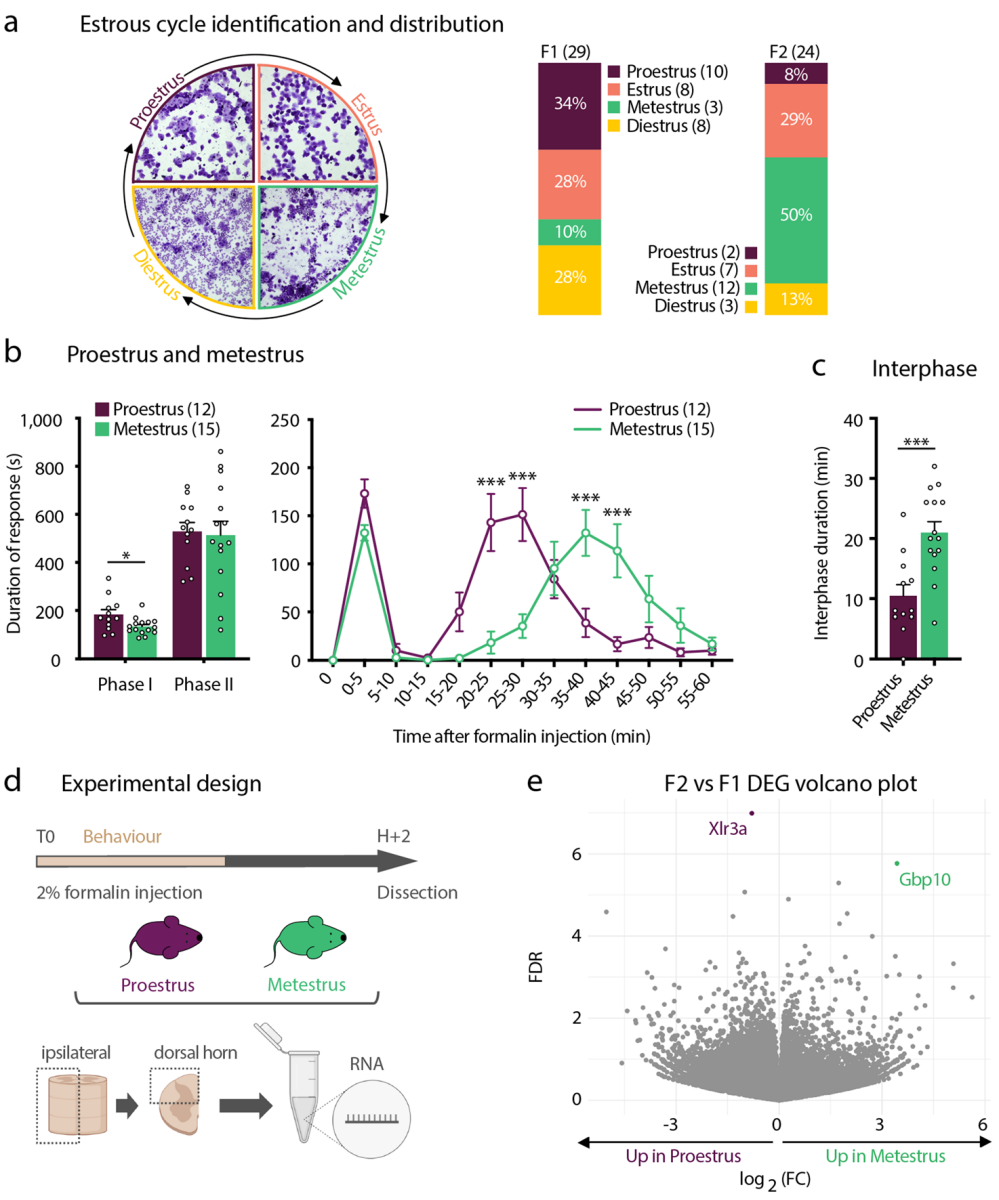
The duration of the interphase in females is influenced by the estrus cycle. (**a**) Vaginal cytology used for the identification of the different stages of the estrus cycle and the percentage of females in proestrus, estrus, metestrus and diestrus in F1 and F2 subgroups. (**b**) Cumulative duration (left) and time-course (right) of formalin-evoked nocifensive behavior (in seconds, s) in females in proestrus (n = 12) as compared to females in metestrus (n = 15). Data are presented as mean ± SEM. (*p < 0.05; ***p < 0.001). (**c**) Duration of the interphase (in minutes, min) in females in proestrus (n = 12) and females in metestrus (n = 15). Data are presented as mean ± SEM. (***p < 0.001). (**d**) Illustration of the experimental design for RNA-Seq preformed on the dorsal horn of the spinal cord (DHSC) of proestrus (n = 6) and metestrus (n = 6) females following formalin injection. (**e**) Volcano-plot representation of the RNA-Seq data, highlighting the transcripts enriched in proestrus females and those in metestrus females (FDR5).

We next searched for the biological underpinnings underlying the differences in formalin-evoked pain behaviors in females. The interphase of the formalin test is mainly thought to reflect the recruitment of endogenous inhibitory systems. One possible explanation is that the variations in the levels of circulating gonadal hormones (estrogen and progesterone) across the estrus cycle, influence the strength of endogenous inhibition recruited during the interphase^19^. We thus determined the stage of the estrus cycle of each of the females analyzed in Fig. 1, right after the formalin test. We found that metestrus females were under-represented in the F1 group (n = 3/29), while F2 group contained only a very small fraction of females in proestrus (n = 2/24) (Fig. 2a). Additionally, when analyzed with respect to their estrus cycle and independently of their belonging to F1 or F2 groups, proestrus females exhibited a F1-like behavior, while females in metestrus displayed a F2-like behavior, with respectively short and long-lasting interphases (Fig. 2b,c). We also observed that females in metestrus exhibited less formalin-evoked pain as compared to females in proestrus, in the first phase of the test (Fig. 2b). Mice that were in estrus and diestrus had an intermediate response to formalin and could be found in both groups (Fig. S1). Hormonal fluctuations across the estrus cycle have been recently shown to affect the transcriptional activity of several genes in the hippocampus, including genes involved in the regulation of neural activity and behavior^22^. We thus further asked if the differences in formalin-evoked behaviors observed in F1/proestrus and F2/metestrus could be also explained by differential gene expression in the DHSC. To this aim, we performed unbiased RNA sequencing (RNA-Seq) on ipsilateral DHSC tissues from F1/proestrus (n = 6) and F2/metestrus females (n = 6), at 2 h (2H) post-formalin, a time-point we estimated will include early-induced genes in response to formalin (Fig. 2d,e).

Comparative analysis with a false discovery rate of 5 (FRD5) revealed only 2 differentially-expressed genes (DEG) between F1/proestrus and F2/metestrus (Fig. 2e). X-linked lymphocyte regulated 3a (*Xlr3a*) was up-regulated in F1/proestrus females with a 1,79-fold-change (FC) as compared to F2/metestrus females. Guanylate-binding protein 10 (*Gbp10*) transcripts were found about 10 times more abundant in F2/metestrus females as compared to F1/proestrus. Both *Xlr3a* and *Gbp10* are described to be part of the immune system^23,24^, potentially suggesting a differential immune status in F1/proestrus and F2/metestrus females DHSC. These data demonstrate that the transcriptional content of the DHSC in formalin-injected F1/proestrus and F2/metestrus females is highly similar. They also put forward a preponderant contribution of the estrus cycle in the post-transcriptional modulation of the mechanisms regulating the duration of the interphase.

### Sex-related transcriptional differences in the ipsilateral DHSC following formalin administration

**Figure 3:**
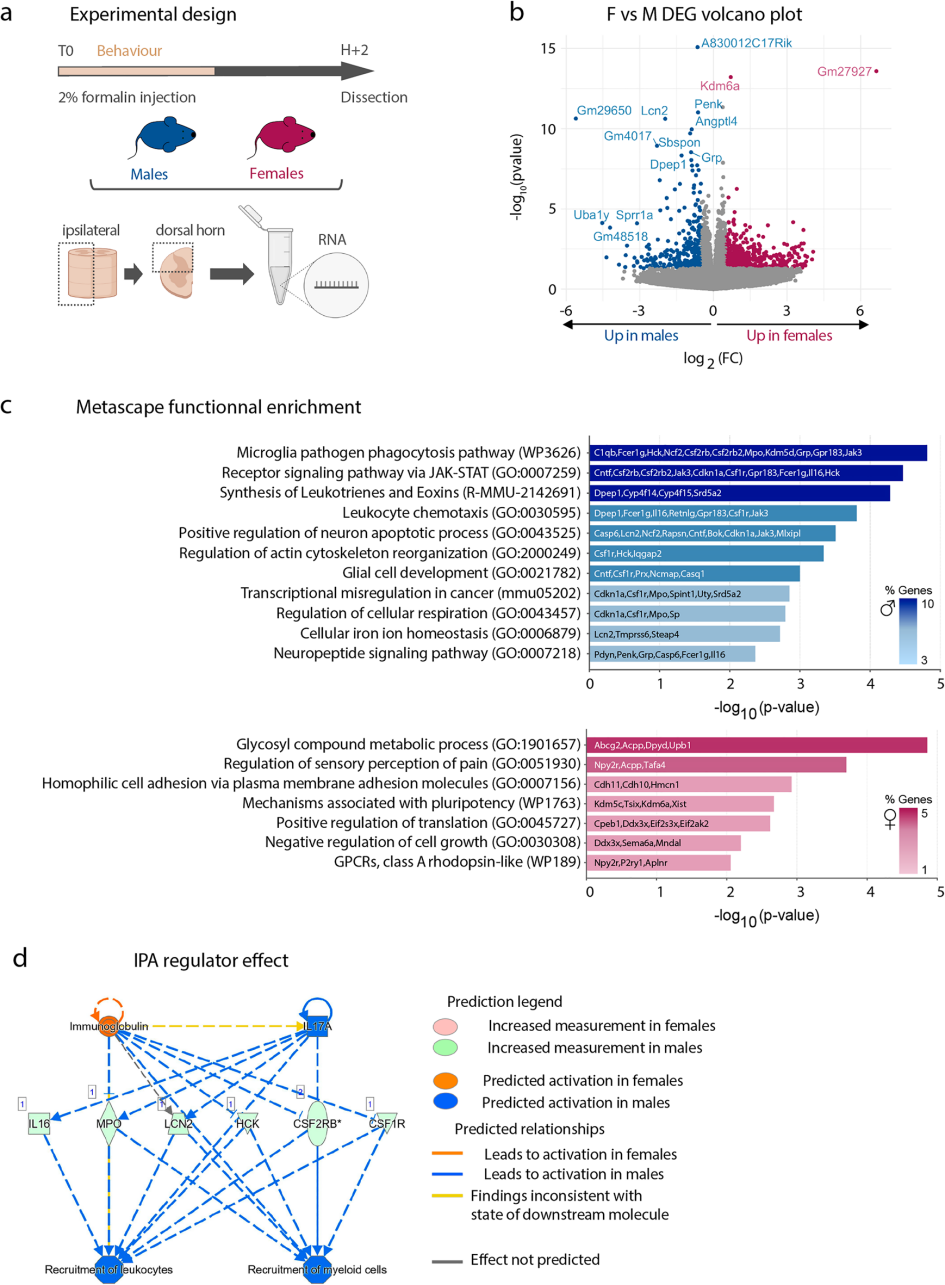
Sex-related transcriptional differences in the ipsilateral DHSC following formalin administration. (**a**) Illustration of the experimental design for RNA-Seq preformed on the dorsal horn of the spinal cord (DHSC) of males (n = 6) and females (n = 12, including n = 6 F1/proestrus and n = 6 F2/metestrus) following formalin injection. (**b**) Volcano-plot representation of the RNA-Seq identified DEG between males and females (FDR5). For a matter of representation, the following DEG were not represented on the volcano plot: *Xist*, *Tsix*, *Gm2223* for females and *Kdm5d*, *Eif2s3y*, *Uty*, *Ddx3y* for males. (**c**) Metascape functional enrichment of transcripts enriched in males and those enriched in females. (**d**) IPA disease and function prediction showing clusters of male-enriched transcripts associated with immune response.

Several studies report that even in absence of quantitative sex differences in pain behavior, there are inherent male–female differences in the molecular and cellular mechanisms underlying pain processing, including in formalin pain^7,20,25^. Thus, we asked whether there were differences in the transcriptional content of the ipsilateral DHSC between males and females, 2H post-formalin injection. Towards this aim, we compared the transcriptional profiles of all the female samples (i.e. F1/proestrus and F2/metestrus females were not analyzed separately, they were combined to be analyzed as a single condition, n = 12) to male samples (n = 6). Using the same RNA-Seq approach as above-described, we found 158 DEG between sexes with FDR of 5, among which 57 were up-regulated in females as compared to males and 101 in males as compared to females (Fig. 3a,b and Table S1). As expected when comparing female versus male transcriptomes, the top-ranking DEG were X and Y-linked genes (*Xist* and *Tsix*, as well as *Uty*, *Kdm5d*, *Eif2s3y* and *Ddx3y*) (Table S1). Functional analysis using Metascape software^26^ revealed different pathways enriched in each sex (Fig. 3c). For instance, female DEG were enriched for transcripts involved in glycosyl compound metabolic regulation, in the sensory regulation of pain and in the positive regulation of translation. Male DEG were enriched for transcripts involved in microglial activity and development, as well as in leukocyte chemotaxis (Fig. 3c). We further used Ingenuity Patway Analysis (IPA) software to interpret the female- and male-enriched genes in the context of biological processes, pathways and networks (Table S2). In line with the Metascape analysis, IPA revealed a significant enrichment of male-DEG involved in the recruitment of leukocytes and recruitment of myeloid cells (Fig. 3d). These genes included macrophages/microglial-associated transcripts such as colony stimulating factor receptor 1 and 2b (*Csf1r*, *Csf2rb*), Interleukin-16 (*Il-16*), as well as genes associated with neutrophil function such as myeloperoxidase (*Mpo*) and lipocalin2 (*Lcn2*). Thus, this analysis further highlights a potentially male-specific spinal neuro-immune regulation of formalin evoked pain, possibly involving microglia, as previously reported^20^, but also neutrophils.

Taken together, our results underscore qualitative sex-related differences in the molecular mechanisms associated with neuronal and neuro-immune regulation in formalin-evoked pain.

### Formalin triggers LCN2-positive neutrophils recruitment to the pia layer of spinal meninges, preferentially in males

**Figure 4:**
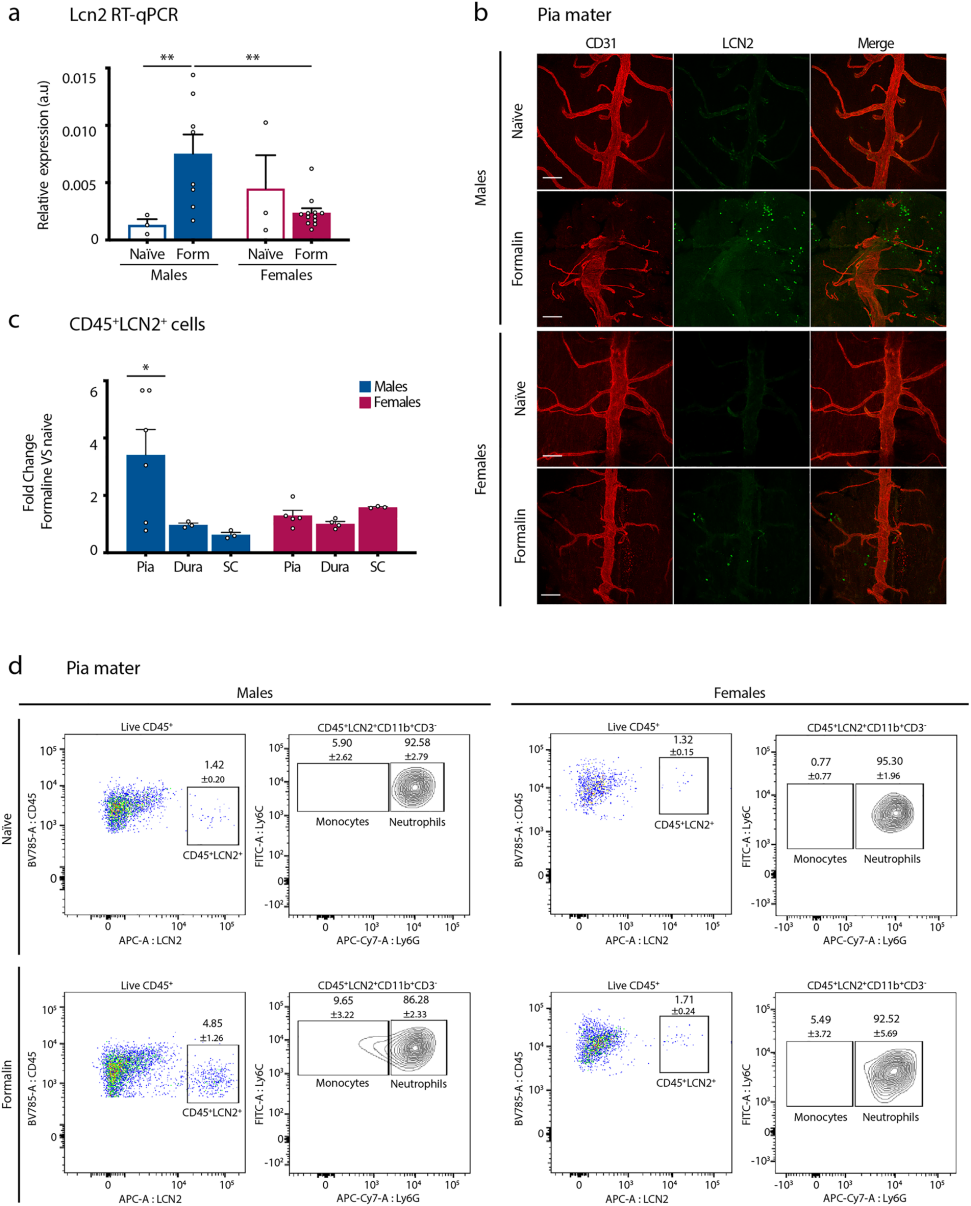
Formalin triggers LCN2-positive neutrophils infiltration in the pia layer of spinal meninges, preferentially in males. (**a**) RT-qPCR analysis of *Lcn2* expression levels in the ipsilateral DHSC of naïve males (n = 3) and free-cycling females (n = 3) and formalin-injected males (n = 8) and free-cycling females (n = 12). Data are presented as mean of relative expression normalized to β-actin and expressed in arbitrary units (a.u) ± SEM. (**p < 0.01). (**b**) Whole-mount immunostaining on L3-L5 segments of the DHSC with anti-CD31 (red) and anti-LCN2 (green) antibodies, of naïve and formalin-injected males and free-cycling females. The tissue is oriented to allow visualization of the dorsal pia mater. Representative of 2 to 3 independent experiments. Scale bars: 100 µm. (**c**) Fold change between formalin and naive conditions of the percentages of CD45^+^LCN2^+^ cells in the pia and dura maters and in the ipsilateral SC in males and in free-cycling females, naïve and 2H after formalin injection. Data are expressed as mean ± SEM calculated from 3 to 6 independent samples. (*p < 0.05). (**d**) Flow cytometry analysis of CD45^+^LCN2^+^ cells in the pia mater of naive and formalin-injected males and free-cycling females. From left to right are shown: the percentages of CD45^+^ LCN2^+^ cells within live CD45^+^ cells, the percentages of Ly6C^+^Ly6G^+^ (LCN2^+^ neutrophils) and Ly6C^+^Ly6G^-^ (LCN2^+^ monocytes) in the CD45^+^LCN2^+^CD11b^+^CD3^-^ gate. Data are expressed as mean ± SEM.

Given the growing interest in sex-specific immune regulation of injury-induced pain^7^, we decided to further explore this aspect in our model. In particular, we were intrigued by the fivefold up-regulation of *Lcn2*-encoding transcripts within the ipsilateral DHSC of males as compared to females (Table S1). Of note, *Lcn2* expression levels were not dependent on the estrus cycle, as we did not observe differences in the transcripts per million (TPM) between F1/proestrus and F2/metestrus females (Fig. S2). LCN2, also known as Neutrophil Gelatinase-Associated Lipocalin (NGAL), is an acute phase protein, reported to be up-regulated and secreted by a wide range of cell types (including neutrophils, monocytes, reactive astrocytes, activated microglia, neurons and endothelial cells) in response to tissue insults^27^. LCN2 has been reported to participate to the pathogenesis of neuropathic and inflammatory pain and to contribute to the amplitude of pain in the second phase of the formalin test^28,29^. Importantly, so far, the role of LCN2 in pain was demonstrated in males only, and our RNA-Seq data pointed towards a sex-predominant mode of action of LCN2, at least in the formalin pain model. Consistent with this hypothesis, 2H post formalin administration, *Lcn2* mRNA was significantly up-regulated in males ipsilateral DHSC as compared to naïve condition, while this was not the case for females (Fig. 4a). In addition, we confirmed that there were no significant changes in *Lcn2* expression levels across the estrus cycle in formalin-injected females, although we observed higher variability in estrus females (Fig. S2). Therefore, all the following experiments were carried on free-cycling females. To determine the identity of the cell types in which such up-regulation occurred, we performed immunolabelling of male spinal cord transversal sections harvested 2H post-formalin. Our results showed that in the DHSC, LCN2 expression occurred in small round cells that were located in CD31^+^ blood vessels, likely irrigating the pia mater of spinal meninges (Fig. S3). Further immunostaining on floating DHSC whole tissue confirmed an increase in the number of these small round LCN2-expressing cells in male, but not female pia mater, that was observed at 2H post-formalin (Fig. 4b). Because, given their shape and size we thought that these LCN2-expressing cells belonged to the immune compartment, we used flow cytometry to gain insight in their identity. We first showed that we could use intracellular staining to accurately detect LCN2-expressing cells with flow cytometry (Fig. S4). We next performed flow cytometry analysis of cell suspensions obtained from the pia mater, dura mater and ipsilateral L3-L5 segments of the SC of both males and females either naive or 2H post-formalin. In all these tissues, the vast majority of LCN2^+^ cells were also positive for leucocyte-marker CD45, confirming that these are immune cells (Fig. S4). Formalin injected males exhibited a significant increase in the percentage CD45^+^LCN2^+^ cells as compared to naïve animals, in the pia mater only (Fig. 4c). Among these CD45^+^LCN2^+^ cells, 96.94 ± 1.08 were CD11b^+^CD3^−^ and the vast majority of them co-expressed Ly6G, demonstrating that they are neutrophils (Fig. 4d). Additionally, the percentage of neutrophils in male pia mater was significantly increased following formalin, and the vast majority of them expressed LCN2 (Fig. S5). The remaining CD45^+^LCN2^+^CD11b^+^CD3^−^Ly6G^−^ ones expressed Ly6C, showing that they belong to monocytic lineage (Fig. 4d). Importantly, formalin did not induce the above-described increase in the percentage of CD45.2^+^LCN2^+^ cells or neutrophils in any of the female tissues analyzed, including pia mater (Figs. 4c,d and S5). Together, these results demonstrate an un-anticipated male-preponderant recruitment of LCN2-expressing neutrophils in the pia mater of the spinal meninges, in response to formalin-evoked pain.

## Discussion

In this study we report sex and estrus-cycle related differences in behavioral response to formalin-evoked pain. We also highlight that formalin-evoked pain is accompanied by sex-related differential gene expression in the DHSC, marked by a male-preponderant enrichment in genes participating to the neuro-immune modulation of pain. Finally, we show that formalin injection promotes the recruitment of LCN2-expressing neutrophils in the pia mater of spinal leptomeninges, preferentially in males. Sex differences in formalin-evoked pain have been documented in both rats and mice, with females exhibiting increased pain behavior as compared to males^10–13,17^. In our study, we did not observe such differences in the amplitude of formalin-evoked pain behavior when we compared the group of male mice with free-cycling females (Fig. 1c). Our data include unusual high numbers of individuals per sex (24 males and 53 females), thus we are confident of the power of our statistical analysis. Differences with what previously reported are very likely to be explained by our experimental setting^21^. What was striking in our study, was that female mice displayed two different types of behaviors in response to formalin, marked by significant differences in the duration of the interphase: F1 females had a short-lasting interphase, while F2 females exhibited a much longer lasting interphase (Fig. 1d–f). We further showed that the duration of the interphase in females was influenced by the stage of females’ estrus cycle, rather than by the transcriptional content of the DHSC (Figs. 2 and S1). The fluctuations in female steroid hormones across the estrus cycle have been reported to influence behavior, including pain^8,22,30,31^. However, it is still unclear to what extent cycling sex hormones influence the sensitivity to experimental pain in both humans and rodents^12,32–35^. Several studies report that in female rats, nociceptive sensitivity is increased during proestrus and is reduced during metestrus/diestrus^8,31,34,36,37^. Moreover, pain symptoms in women with chronic pain change with the fluctuations of gonadal hormones across the menstrual cycle^1,3,38^. Regarding formalin-evoked pain, Kim et al., and Vincler et al., reported that the estrus cycle does not influence the amplitude of formalin-evoked pain in the first and in the second phase of the test, respectively in mice and rats^12,35^. However, studies on ovariectomized female rats have shown that both estrogens and progesterone regulate the intensity of formalin-evoked pain^17,39^. We found striking differences in the duration of the interphase of the formalin test amongst females, with females in proestrus and in metestrus exhibiting respectively a short and a long-lasting interphase (Fig. 2b). Importantly, neither Kim and colleagues, nor Vincler and colleagues have evaluated the pain behaviors during the interphase^12,35^. Thus, our findings suggest that cycling female hormones regulate the duration of the interphase of the formalin test (Fig. 2b). The interphase of the formalin test represents the recruitment of spinal endogenous inhibition^16,40^. One important regulator of the interphase of the formalin test is the ionotropic gamma aminobutyric acid (GABA)-ergic system^40^. Our data suggest that the low estradiol levels and the rising levels of progesterone that characterize females in metestrus potentiate the endogenous spinal inhibition recruited during the interphase. Consistent with this hypothesis, the progesterone metabolite derivate allopregnanolone has been shown to be a potent positive allosteric modulator of spinal ionotropic GABAergic receptors^41^ and systemic administration of allopregnanolone reduced formalin-evoked pain in rats^42^.

The other finding of this study is that there are significant differences in the transcriptional content of the DHSC in formalin-exposed males and females (Fig. 3). One of the most striking difference is underlined by an over-representation of transcripts involved in microglia activity and the recruitment of immune cells, in males (Fig. 3c,d). We acknowledge that our experimental design does not include naive controls, thus the sets of DEG identified here contain both transcripts that are differentially expressed at steady-state and in response to formalin injection. Recent studies demonstrate fundamental sex differences in neuro-immune interactions in the spinal cord, in particular regarding the contribution of spinal microglia to pathological pain^7^. It has been shown that spinal microglia contribute more prominently in males than in females to inflammatory pain and to chronic neuropathic pain^43–45^. Most recent reports based on single-cell RNA-Seq analysis of spinal microglia demonstrate a robust inflammatory response predominantly in male microglia at early time-points post-neuropathic injury^46^. Hence, our RNA-Seq data, even if performed on total DHSC tissue, which contains several cell types, support a predominant activation of microglia in formalin-injected males (Fig. 3c). Consistently, pharmacological inhibition of spinal microglia has a more pronounced effect in decreasing formalin-evoked pain in males as compared to females^20^.

Finally, our data indicate that formalin promotes the recruitment of LCN2-expressing neutrophils in spinal leptomeninges, preferentially in males as compared to females (Fig. 4). Meninges of the central nervous system harbor very diverse subsets of immune cells, which impact on its function both in physiological and pathological conditions^47^. The contribution of spinal meningeal immune system to distinct pain conditions is currently emerging^48,49^. Here, we report for the first time a sex-dependent recruitment of LCN2^+^ neutrophils in spinal leptomeninges in the formalin pain model (Fig. 4b–d). Neutrophils are the most abundant circulating immune cell types in the blood, rapidly recruited at inflammatory sites. Neutrophils are a heterogenous pool of immune cells, as they exist in various maturation states, and LCN2 is preferentially expressed in immature neutrophils^50,51^. Sex differences in neutrophil biology have been acknowledged in both humans and mice^51–53^. Neutrophils from reproductive-aged males are more immature than their female counterparts^51,52^ and female mice recruit fewer neutrophils upon detection of an inflammatory stimulus^53^. Consistent with this, we did not observe that formalin induced an increase in the total population of neutrophils (LCN2^+^ or LCN2^−^) in either of the female tissues analyzed (Fig. S4). Thus, our data fit in this immunological literature which shows that even if neutrophils are in a more immature state, they are more abundantly recruited at inflammatory sites, in males. With respect to its function in pain biology, LCN2 participates to pain amplification in inflammatory (including formalin pain) and neuropathic pain models, via both peripheral and spinal mechanisms^28,29,54^. LCN2 receptor is widely expressed in spinal microglia and astrocytes^27^ in which it induces inflammatory cytokine expression. On this basis and on the basis of our data, we propose that LCN2 release from pia neutrophils may contribute to formalin-evoked spinal sensitization mechanisms, preferentially in males.

In conclusion, our data consolidate the contribution of female estrus cycle to pain perception and provide evidence supporting a sex-specific immune regulation of formalin-evoked pain.

## Materials and methods

### Mice

C57BL/6J males and females were purchased from Janvier Labs. Mice were maintained under standard housing conditions (22 °C, 40% humidity, 12 h light cycles, and free access to food and water). All experiments were conducted on adult mice (8 to 12 weeks). Special effort was made to minimize the number as well as the stress and suffering of mice used in this study.

### Ethics declaration

The authors confirm that all experimental protocols were approved by the French Ministry of Research and Innovation (APAFIS 2021062313194207-V1 #30046). All methods were carried out in accordance with European Union recommendations for animal experimentation (EU0221). The authors confirm that all experimental procedures were accordance with ARRIVE guidelines.

### Formalin test

Animals were habituated to the experimenter before performing the test. Formalin solution was prepared at 2%, by diluting the 37% formaldehyde stock solution (Fischer Scientific) in phosphate buffer saline (PBS) 1X. Mice were placed individually into Plexiglas chambers, allowed to habituate to the testing environment for 30 min. Mice were weighted and formalin solution was injected subcutaneously in the plantar surface of the left hind paw, at a volume of 8 µL/20 g. The animals were immediately placed individually in observation chambers and then monitored for pain behavior (shaking, licking and biting of the injected paw) for 60 min. The cumulative pain behavior was counted in seconds, per 5 min intervals.

### K-means clustering

The time course of the behavioral response to formalin injection of each mice were partitioned into two clusters per sex using k-means algorithm to reduce data dimensionality. The k-means algorithm treats each single time course segment as a point in 13-dimensional space and groups data into k mutually exclusive clusters by minimizing the centroid distance of observations within clusters and maximizing the distance between clusters.

### Determination of estrus cycle

Right after the formalin test, females’ estrus cycle was determined by gentle repetitive vaginal injection and aspiration of 10 µL distilled water, to collect cells from vaginal wall. Vaginal fluid was deposited on glass slides, allowed to dry and then stained with Crystal Violet (1 min, followed by 2 washes of 2 min each, in water), as previously^55^. Vaginal smears were examined under light microscopy and the types and proportions of the cells present was identified, allowing to determine the stage of the estrus cycle.

### RNA-extraction

Mice were deeply anesthetized with a mix of ketamine/xylazine, L3 to L5 spinal segments were rapidly extracted, the ipsilateral dorsal horn was dissected and RNA was extracted by using RNeasy Micro Kit (Quiagen), according to manufacturer's instructions. For quality control, RNAs were loaded on an RNA PicoChip (Agilent) and processed with 2100 Bioanalyzer system (Agilent technology). High quality RNA (RIN ≥ 9) was used for RNA-Seq and q-RT-PCR.

### RNA-Seq

RNA-Seq was performed on RNA from ipsilateral DHSC of males (n = 6), F1/proestrus (n = 6) and F2/metestrus (n = 6) females, at 2H following formalin injection. RNA-seq libraries were prepared using the TruSeq RNA Sample Preparation Kit (Illumina). All libraries were validated for concentration and fragment size using Agilent DNA1000 chips. Sequencing was performed on a HiSeq 2000 (Illumina), base calling performed using RTA (Illumina) and quality control performed using FastQC (FASTQC, 2010) and RSeQC^56^. Sequences were uniquely mapped to the mm10 genome using Subread (C version 1.4.6-p2) using default values. Reads mapping to gene exons (GRCm38.p4 gene assembly) were counted using featureCounts^57^ (C version 1.4.6-p2). Differential gene expression was performed using exon counts from biological replicates using the DESeq2 BioConductor R package^58^, using a 5% false discovery rate (FDR) cutoff.

### qRT-PCR

RNA obtained from each sample was converted into cDNA using Superscript III Reverse Transcriptase (Invitrogen). Gene expression was assessed by quantitative PCR (qPCR), using qPCR Sybr-Green master mix (ThermoFisher). Samples were run for 40 cycles on a StepOne qPCR apparatus (Applied Biosystems). The relative quantity of transcript encoding each gene was determined by normalization to β-actin using the standard ΔCt method. The following primers were used:

- Lcn2-Fw: ACAACCAGTTCGCCATGGTAT
- Lcn2-Rse: GCTCCTTGGTTCTTCCATACAG
- b-actin-Fw: ACTGTCGAGTCGCGTCCA
- b-actin-Rse: TCGTCATCCATGGCGAACTG

### Immunostaining

#### Immunostaining on transverse SC sections

Mice were deeply anesthetized with a mix of ketamine/xylazine and then transcardially perfused with an ice-cold solution of paraformaldehyde 4% diluted in PBS 1X (4% PFA). Tissues were further fixed for 24 h in ice-cold 4% PFA. Specimens were transferred into a 30% (w/v) sucrose solution for cryoprotection before being frozen in OCT mounting medium.18–20 µm transversal SC cryosections were obtained using a standard cryostat (Leica). For immunostaining, sections were incubated for 1 h at room temperature (RT) with Blocking Buffer (BB) containing PBS 1X supplemented with 10% (vol/vol) donkey serum (Sigma), 3% (weight/vol) bovine albumin (Sigma)—0.4% (vol/vol) Triton X-100, for saturation, then and incubated overnight at 4 °C with primary antibodies diluted in BB. Primary antibodies used in this study are as follows: goat-anti LCN2 (AF1857-SP, R&D systems) and rat anti-CD31 (553370, BD Pharmingen). After three washes with PBS1X of 5 min each, sections were incubated for 1 h at RT with donkey anti-rat and anti-goat respectively coupled with Alexa 488 and Alexa 555 fluorophores (Invitrogen or Molecular probe antibodies), diluted in BB. Tissues were washed (3 times in PBS1X) before being mounted with ImmuMount Reagent.

Image acquisition was performed using AxioImager Z1 (Zeiss) fluorescence microscope and contrast was adjusted using Fiji software.

#### Whole-mount floating immunostaining

Mice were deeply anesthetized with a mix of ketamine/xylazine and then L3 to L5 SC segments were rapidly dissected. Special care was provided to dissect out the arachnoid mater. The dorsal part was further dissected longitudinally and immediately placed in ice-cold PFA4% for an overnight fixation at 4 °C. Tissues were washed with PBS 1X and then saturated with BB for 1 h at room temperature, prior to incubation with goat-anti LCN2 and rat anti-CD31 primary antibodies (2 to 3 h at room temperature). After 3 washings with PBS 1X, of 5 min each, tissues were incubated for 1 h at room temperature with donkey anti-rat and anti-goat respectively coupled with Alexa 488 and Alexa 555 fluorophores. Tissues were washed (3 times in PBS1X) before being mounted on glass slides, the dorsal part containing the pia mater facing upwards, with ImmuMount Reagent.

Image acquisition was performed using LSM-780 confocal microscope (Zeiss) and images were analyzed using Fiji software.

### Flow cytometry

Mice were lethally anesthetized using a mixture of xylazine and ketamine and vertebral column corresponding to the spinal L3–L5 segments was extracted and placed in ice-cold RPMI for further processing. SC was carefully removed from the vertebral column, as well as the dura mater of the spinal meninges. The dorsal and ventral roots of SC were carefully cut-out and the pia mater was separated from the SC. In formalin-injected subjects, ipsilateral SC was harvested and cut into small pieces before being digested using a mixture of collagenase D (2 mg/ml, Roche), DNAseI (50 µg/ml, Roche), diluted in RPMI medium (Gibco), for 1 h at 37 °C. Pia and dura maters underwent the same enzymatic digestion protocol. At the end of the enzymatic digestion, tissues were immediately placed on ice, supplemented with RPMI + 10%FCS and triturated using 21-G needles-equipped syringes. The resulting cell suspensions were filtered on 100 µm filters, washed with RPMI + 10%FCS and centrifugated at 1500 rpm, 4 °C, for 7 min. SC pellets were resuspended in an isotonic 40% Percoll solution, diluted with RPMI and overlaid onto a 80% Percoll solution. Following centrifugation at 2800 rpm, for 30 min at room-temperature, the ring of cells at the 40–80 interface was collected and washed with RPMI + 10% FCS. SC, pia and dura mater pellets were homogenized in FACS buffer (PBS1X supplemented with 5% FCS and 2 mM EDTA, Gibco) and processed for immunostaining. Cell suspensions from brain tissue, prepared using the same protocol as for SC, were included as control.

Cell surface immunostaining was performed as follows: cells were incubated with Mouse BD Fc Block™ (clone 2.4G2, BD bioscience), for 15 min on ice, then they were stained with a mix of fluorophores-labelled antibodies, for 30 min on ice. After washing with FACS buffer, then with PBS1X, death cells were stained using LIVE/DEAD™ Fixable Aquadead Cell Stain Kit for 405 nm excitation (Invitrogen) for 20 min on ice, according to manufacturer’s instructions, then they were fixed and permeabilized using BD Cytofix/Cytoperm Kit (BD bioscience) and were further subjected to intracellular LCN2 staining. Cells were incubated with uncoupled polyclonal goat anti-LCN2 antibodies (AF1857-SP, R&D systems) diluted in Perm-Wash solution (BD), overnight at 4 °C prior to being stained with donkey anti-goat Alexa 647 coupled secondary antibodies (30 min, on ice). Both goat serum and secondary antibody alone performed on brain mononuclear cell suspensions were used as controls for LCN2 intracellular staining. Stained cells were analyzed on a LSRII-UV flow cytometer (BD bioscience) and Diva (BD bioscience) and FlowJo software were used for data analysis.

The antibody cell surface mix contained: Ly6C-FITC (clone AL-21, BD bioscience), NK1.1-PE (clone PK136, BD bioscience), CD3e-PECy5 (clone 145-2C11, BD bioscience), F4/80-PE-CF584 (clone T45-2342, BD bioscience), Ly6G-APC-Cy7 (clone1A8, Biolegend), CX3CR1-BV421 (clone SA011F11, Biolegend), CD11b-BV510 (clone M1/70, BD bioscience) and CD45.2-BV785 (clone 3-F-11, Biolegend).

### Statistical analysis

The number of tested animals is indicated in the figure legends section.

The Shapiro–Wilk test was used to assess data normality. Statistical significance was set to p < 0.05 and assessed using two-way ANOVA followed by Bonferroni's multiple comparisons test (for formalin time course), or multiple unpaired t-test using the Sidak-Bonferroni method (for formalin cumulative time, interphase duration, RT-qPCR data and flow cytometry Fold Change). Statistical analyses were performed with GraphPad Prism 9.0 (GraphPad Software, Inc., San Diego, CA).

## Supplementary Information

Supplementary Information.

## Data Availability

All high throughput sequencing data have been deposited with the Gene Expression Ombudsman (GEO) and are available under the accession number GSE226443.

## References

[1] Riley JL, Robinson ME, Wise EA, Myers CD, Fillingim RB (1998). Sex differences in the perception of noxious experimental stimuli: A meta-analysis. Pain.

[2] Mogil JS (2012). Sex differences in pain and pain inhibition: Multiple explanations of a controversial phenomenon. Nat. Rev. Neurosci..

[3] Fillingim RB, King CD, Ribeiro-Dasilva MC, Rahim-Williams B, Riley JL (2009). Sex, gender, and pain: A review of recent clinical and experimental findings. J. Pain.

[4] Unruh AM (1996). Gender variations in clinical pain experience. Pain.

[5] LeResche L, Mancl LA, Drangsholt MT, Saunders K, Korff MV (2005). Relationship of pain and symptoms to pubertal development in adolescents. Pain.

[6] Lenert ME, Avona A, Garner KM, Barron LR, Burton MD (2021). Sensory neurons, neuroimmunity, and pain modulation by sex hormones. Endocrinology.

[7] Mogil JS (2020). Qualitative sex differences in pain processing: Emerging evidence of a biased literature. Nat. Rev. Neurosci..

[8] Bradshaw H, Miller J, Ling Q, Malsnee K, Ruda MA (2000). Sex differences and phases of the estrous cycle alter the response of spinal cord dynorphin neurons to peripheral inflammation and hyperalgesia. Pain.

[9] Vacca V (2014). Higher pain perception and lack of recovery from neuropathic pain in females: A behavioural, immunohistochemical, and proteomic investigation on sex-related differences in mice. Pain.

[10] Aloisi AM, Albonetti ME, Carli G (1994). Sex differences in the behavioural response to persistent pain in rats. Neurosci. Lett..

[11] Gaumond I, Arsenault P, Marchand S (2002). The role of sex hormones on formalin-induced nociceptive responses. Brain Res..

[12] Kim SJ, Calejesan AA, Li P, Wei F, Zhuo M (1999). Sex differences in late behavioral response to subcutaneous formalin injection in mice. Brain Res..

[13] Hagiwara H (2021). Sex differences in pain-induced modulation of corticotropin-releasing hormone neurons in the dorsolateral part of the stria terminalis in mice. Brain Res..

[14] Dubuisson D, Dennis SG (1977). The formalin test: A quantitative study of the analgesic effects of morphine, meperidine, and brain stem stimulation in rats and cats. Pain.

[15] Tjølsen A, Berge O-G, Hunskaar S, Rosland JH, Hole K (1992). The formalin test: An evaluation of the method. Pain.

[16] Henry JL, Yashpal K, Pitcher GM, Coderre TJ (1999). Physiological evidence that the ‘interphase’ in the formalin test is due to active inhibition. Pain.

[17] Gaumond I, Arsenault P, Marchand S (2005). Specificity of female and male sex hormones on excitatory and inhibitory phases of formalin-induced nociceptive responses. Brain Res..

[18] Nazarian A, Tenayuca JM, Almasarweh F, Armendariz A, Are D (2014). Sex differences in formalin-evoked primary afferent release of substance P: Sex differences in substance P release. Eur. J. Pain.

[19] Gaumond I, Spooner M-F, Marchand S (2007). Sex differences in opioid-mediated pain inhibitory mechanisms during the interphase in the formalin test. Neuroscience.

[20] Chen G, Luo X, Qadri MY, Berta T, Ji R-R (2018). Sex-dependent glial signaling in pathological pain: Distinct roles of spinal microglia and astrocytes. Neurosci. Bull..

[21] Bohic M (2020). Loss of bhlha9 impairs thermotaxis and formalin-evoked pain in a sexually dimorphic manner. Cell Rep..

[22] Jaric I, Rocks D, Greally JM, Suzuki M, Kundakovic M (2019). Chromatin organization in the female mouse brain fluctuates across the oestrous cycle. Nat. Commun..

[23] Bergsagel PL, Timblin CR, Kozak CA, Kuehl WM (1994). Sequence and expression of murine cDNAs encoding Xlr3a and Xlr3b, defining a new X-linked lymphocyte-regulated Xlr gene subfamily. Gene.

[24] Kim B-H (2011). A family of IFN-γ–inducible 65-kD GTPases protects against bacterial infection. Science.

[25] Taves S (2016). Spinal inhibition of p38 MAP kinase reduces inflammatory and neuropathic pain in male but not female mice: Sex-dependent microglial signaling in the spinal cord. Brain. Behav. Immun..

[26] Zhou Y (2019). Metascape provides a biologist-oriented resource for the analysis of systems-level datasets. Nat. Commun..

[27] Jha MK (2015). Diverse functional roles of lipocalin-2 in the central nervous system. Neurosci. Biobehav. Rev..

[28] Jeon S (2013). Role of lipocalin-2-chemokine axis in the development of neuropathic pain following peripheral nerve injury. J. Biol. Chem..

[29] Jha MK (2014). The pivotal role played by lipocalin-2 in chronic inflammatory pain. Exp. Neurol..

[30] Kaur S (2018). Sex differences and estrous cycle effects of peripheral serotonin-evoked rodent pain behaviors. Neuroscience.

[31] Kayser V, Berkley KJ, Keita H, Gautron M, Guilbaud G (1996). Estrous and sex variations in vocalization thresholds to hindpaw and tail pressure stimulation in the rat. Brain Res..

[32] Klatzkin RR, Mechlin B, Girdler SS (2010). Menstrual cycle phase does not influence gender differences in experimental pain sensitivity. Eur. J. Pain.

[33] Iacovides S, Avidon I, Baker FC (2015). Does pain vary across the menstrual cycle? A review. Eur. J. Pain.

[34] Martínez-Gómez M, Cruz Y, Salas M, Hudson R, Pacheco P (1994). Assessing pain threshold in the rat: Changes with estrus and time of day. Physiol. Behav..

[35] Vincler M, Maixner W, Vierck CJ, Light AR (2001). Estrous cycle modulation of nociceptive behaviors elicited by electrical stimulation and formalin. Pharmacol. Biochem. Behav..

[36] Drury RA, Gold RM (1978). Differential Effects of Ovarian Hormones on Reactivity to Electric Footshock in the Rat. Physiol. Behav..

[37] Leer MN, Bradbury A, Maloney JC, Stewart CN (1988). Elevated shock threshold in sexually receptive female rats. Physiol. Behav..

[38] Hassan S, Muere A, Einstein G (2014). Ovarian hormones and chronic pain: A comprehensive review. Pain.

[39] Kuba T (2006). Estradiol and progesterone differentially regulate formalin-induced nociception in ovariectomized female rats. Horm. Behav..

[40] Kaneko M, Hammond DL (1997). Role of spinal gamma-aminobutyric acidA receptors in formalin-induced nociception in the rat. J. Pharmacol. Exp. Ther..

[41] Poisbeau P (2005). Inflammatory pain upregulates spinal inhibition via endogenous neurosteroid production. J. Neurosci..

[42] Ocvirk R, Pearson Murphy BE, Franklin KBJ, Abbott FV (2008). Antinociceptive profile of ring A-reduced progesterone metabolites in the formalin test. Pain.

[43] Paige C, Maruthy GB, Mejia G, Dussor G, Price T (2018). Spinal inhibition of P2XR or p38 signaling disrupts hyperalgesic priming in male, but not female, mice. Neuroscience.

[44] Sorge RE (2015). Different immune cells mediate mechanical pain hypersensitivity in male and female mice. Nat. Neurosci..

[45] Mapplebeck JCS (2018). Microglial P2X4R-evoked pain hypersensitivity is sexually dimorphic in rats. Pain.

[46] Tansley S (2022). Single-cell RNA sequencing reveals time- and sex-specific responses of mouse spinal cord microglia to peripheral nerve injury and links ApoE to chronic pain. Nat. Commun..

[47] Alves de Lima K, Rustenhoven J, Kipnis J (2020). Meningeal Immunity and Its Function in Maintenance of the Central Nervous System in Health and Disease. Annu. Rev. Immunol..

[48] Kuhn JA (2021). Regulatory T-cells inhibit microglia-induced pain hypersensitivity in female mice. Elife.

[49] Maganin AGM (2022). Meningeal dendritic cells drive neuropathic pain through elevation of the kynurenine metabolic pathway in mice. J. Clin. Investig..

[50] Evrard M (2018). Developmental analysis of bone marrow neutrophils reveals populations specialized in expansion, trafficking, and effector functions. Immunity.

[51] Gupta S (2020). Sex differences in neutrophil biology modulate response to type I interferons and immunometabolism. Proc. Natl. Acad. Sci..

[52] Blazkova J (2017). Multicenter systems analysis of human blood reveals immature neutrophils in males and during pregnancy. J. Immunol..

[53] Kay E, Gomez-Garcia L, Woodfin A, Scotland RS, Whiteford JR (2015). Sexual dimorphisms in leukocyte trafficking in a mouse peritonitis model. J. Leukoc. Biol..

[54] Jha MK, Jeon S, Jin M, Lee W-H, Suk K (2013). Acute phase protein lipocalin-2 is associated with formalin-induced nociception and pathological pain. Immune Netw..

[55] McLean AC, Valenzuela N, Fai S, Bennett SAL (2012). Performing vaginal lavage, crystal violet staining, and vaginal cytological evaluation for mouse estrous cycle staging identification. J. Vis. Exp..

[56] Wang L, Wang S, Li W (2012). RSeQC: Quality control of RNA-seq experiments. Bioinformatics.

[57] Liao Y, Smyth GK, Shi W (2014). featureCounts: An efficient general purpose program for assigning sequence reads to genomic features. Bioinformatics.

[58] Love MI, Huber W, Anders S (2014). Moderated estimation of fold change and dispersion for RNA-seq data with DESeq2. Genome Biol..

