# Supplementary material for: Formalin-evoked pain triggers sex-specific behavior and spinal immune response
Source: Sci Rep. 2023 Jun 12;13:9515. doi: 10.1038/s41598-023-36245-7 (PMC10261048; doi:10.1038/s41598-023-36245-7)
Supplement: Supplementary file 1 — Supplementary Information. [file 41598_2023_36245_MOESM1_ESM.pdf]

Supplemental Information

Content:

Supplemental Figures S1-5 and Supplemental Figures Legends

Supplemental Table S1

Supplemental Table S2

Figure S1

a

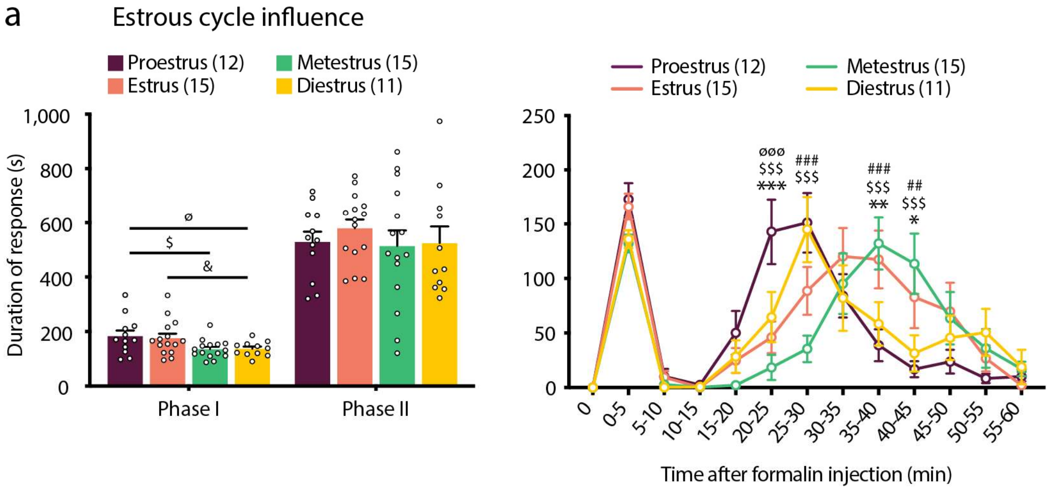

Estrous cycle influence
Proestrus (12)
Estrus (15)
Metestrus (15)
Diestrus (11)
Duration of response (s)
1,000
800
600
400
200
0
ø
$
&
Phase I
Phase II
250
200
150
100
50
0
øøø
$$$
***
###
$$$
###
$$$
**
##
$$$
*
0
0-5
5-10
10-15
15-20
20-25
25-30
30-35
35-40
40-45
45-50
50-55
55-60
Time after formalin injection (min)

Figure S2

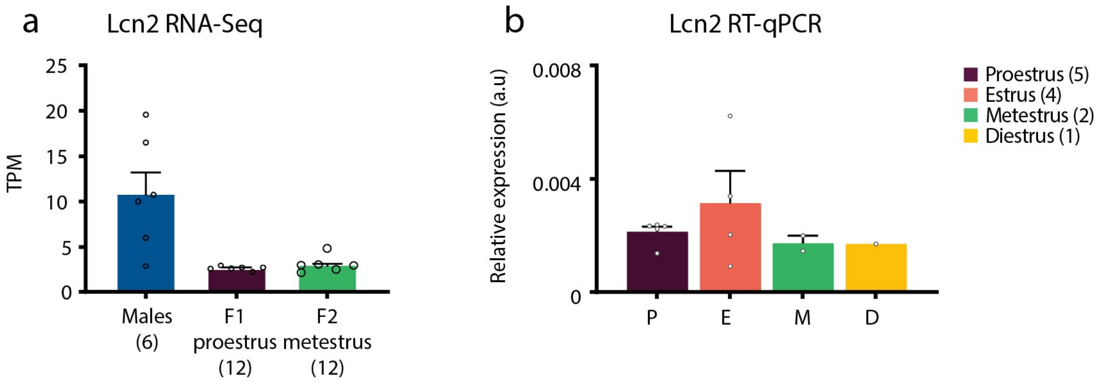
a
Lcn2 RNA-Seq
TPM
25
20
15
10
5
0
Males
(6)
F1
proestrus
(12)
F2
metestrus
(12)
b
Lcn2 RT-qPCR
Relative expression (a.u)
0.008
0.004
0
P
E
M
D
Proestrus (5)
Estrus (4)
Metestrus (2)
Diestrus (1)

Figure S3

a Dorsal horn of the spinal cord b Dorsal median sulcus

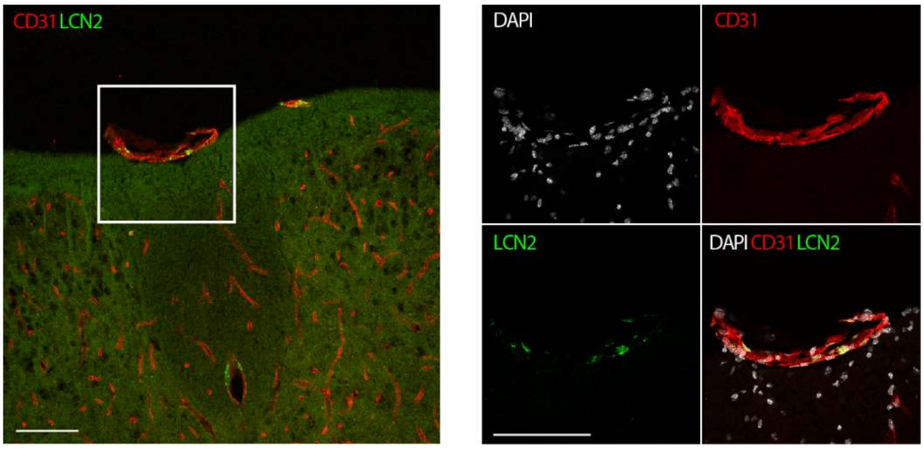

Figure S4

a Gating strategy

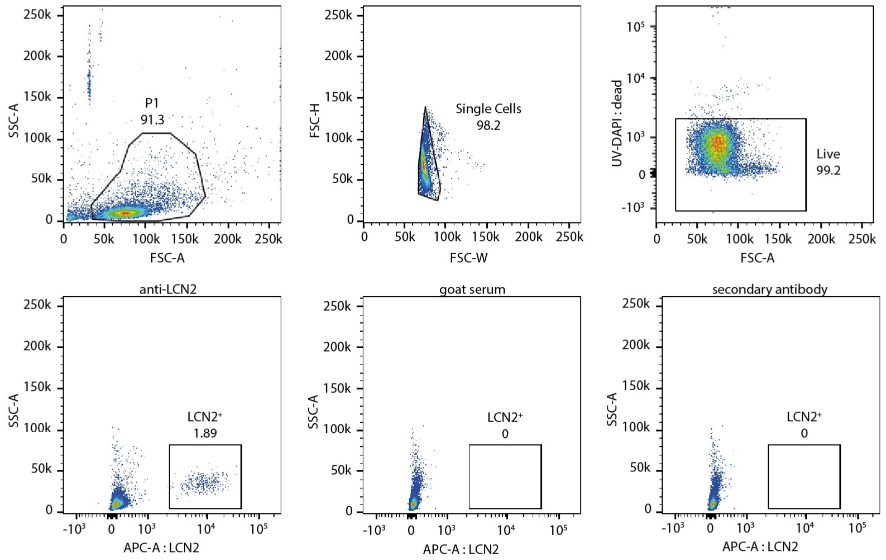

b Backgating strategy

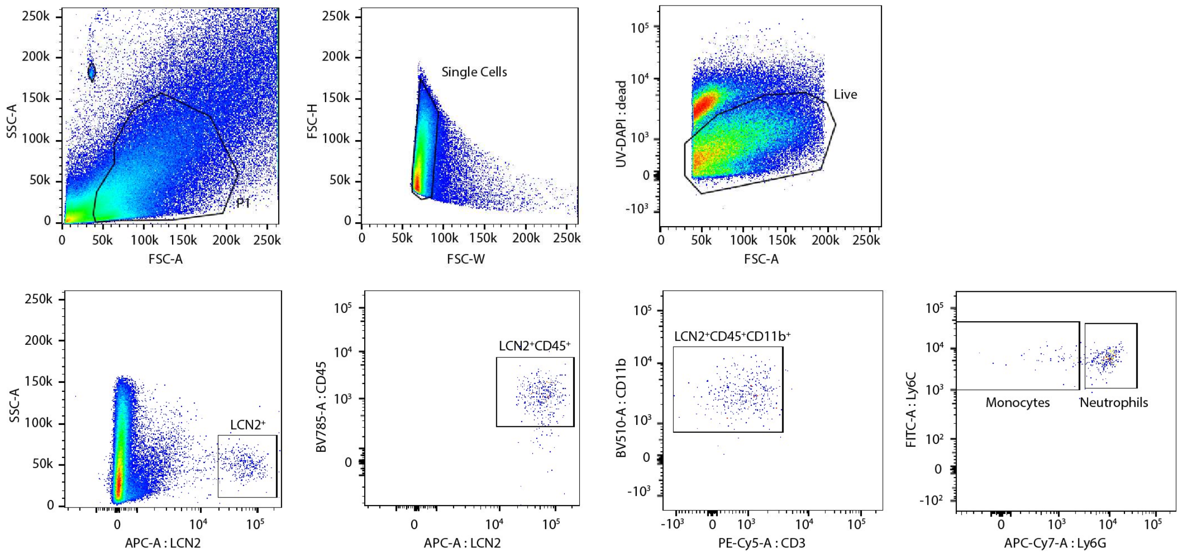

Supplemental Figure Legends

Figure S1: Estrus cycle influence on female formalin-evoked pain behavior

Cumulative duration (left) and time-course (right) of formalin-evoked nocifensive behavior (in seconds, s) in females in proestrus (n=12), estrus (n=15), metestrus (n=15) and diestrus (n=11). Data are presented as mean ± SEM. (proestrus-estrus: * $p < 0.05$; ** $p < 0.01$; *** $p < 0.001$) (proestrus-metestrus: $ $p < 0.05$; $$$ $p < 0.001$) (proestrus-diestrus: ø $p < 0.05$; øøø $p < 0.001$) (estrus-diestrus: & $p < 0.05$) (metestrus-diestrus: ## $p < 0.01$; ### $p < 0.001$)

Figure S2: *Lcn2* expression in the dorsal horn of the spinal cord (DHSC) in formalin-injected males and in females across the estrus cycle

(a) *Lcn2* expression levels in Transcripts per Million (TPM) obtained by RNA-Seq in the ipsilateral DHSC of males (n=6), F1/proestrus females (n=6) and F2/metestrus females (n=6), harvested 2H post-formalin injection in the hind-paw. (b) *Lcn2* expression levels normalized to β-actin, determined by RT-qPCR in the ipsilateral DHSC of females in P: proestrus (n= 5), E: estrus (n=4), M: metestrus (n=2) and D: diestrus (n=1), at 2H post formalin injection into the hind-paw.

Figure S3: LCN2 immunostaining on transversal spinal cord cryosections

(a-b) Immunostaining on SC cryosections from formalin-injected male harvested 2H post-formalin, with anti-CD31 (red) and anti-LCN2 (green) antibodies. (a) Shows the dorsal horn with a pia vessel attached. (b). Zoom on the corresponding inset.

Figure S4: Validation of intracellular LCN2 staining in flow cytometry and determination of the identity of $LCN2^{+}$ cells in the pia mater of the SC

(a) FACS analysis of brain cell suspension used to validate the accuracy of intracellular LCN2 staining. Upper panels show the gating strategy. Cells were gated according to size and structure; doublets and dead cells were eliminated. Lower panel shows the total fluorescence in the APC channel using anti-LCN2 purified antibodies (left), goat serum (middle) or none (right), followed by donkey-anti goat Alexa 647 coupled secondary antibody.

(b) Back-gating strategy used to determine the identity of $LCN2^+$ cells in the pia mater. Upper panels: cells were gated according to size and structure and doublets and dead cells were eliminated. Lower panels: total $LCN2^+$ cells were gated (left) and the population of $LCN2^+CD45^+$ cells was further gated (middle). Within this gate, the population of $LCN2^+CD45^+CD11b^+$ was gated and was further subdivided into $Ly6G^+Ly6C^+$ cells (neutrophils) and $Ly6G^-Ly6C^+$ cells (monocytes). The sample represented here is the pia mater from formalin-injected males.

Figure S5: Neutrophil recruitment to the pia mater of spinal leptomeninges in naïve and formalin-injected males and females

Shows the percentages of $CD11b^+Ly6G^+$ neutrophils gated on Live/$CD45.2^+CD3^-$ cells in naive and formalin injected males (left) and free cycling females (right). The percentages of $LCN2^+$ neutrophils are also represented. Data are expressed as mean ± SEM. (n=3 to 6 per condition).

Table S1: List of female versus male DEG

Gene ID	Gene name	log2(FC)	FDR
ENSMUSG00000086503	Xist	8.1853	1.87E-169
ENSMUSG00000098743	Gm27927	6.6401	6.29E-11
ENSMUSG00000085715	Tsix	6.5036	7.22E-57
ENSMUSG00000098078	Gm26992	3.2501	1.91E-02
ENSMUSG00000111752	Gm38575	2.2201	2.47E-02
ENSMUSG00000102448	Gm37101	1.4904	2.96E-02
ENSMUSG00000106292	Gm43174	1.3483	4.01E-02
ENSMUSG00000004651	Tyr	1.3015	2.44E-02
ENSMUSG00000063286	Gm8995	0.9520	4.16E-04
ENSMUSG00000032561	Acpp	0.8634	6.31E-03
ENSMUSG00000090272	Mndal	0.8467	2.35E-02
ENSMUSG00000028037	Ifi44	0.8309	1.48E-02
ENSMUSG00000033427	Upb1	0.8114	1.87E-02
ENSMUSG00000037369	Kdm6a	0.7056	1.35E-10
ENSMUSG00000041596	Nlrp5-ps	0.6848	4.47E-02
ENSMUSG00000079297	Gm2223	0.6649	2.14E-55
ENSMUSG00000066842	Hmcn1	0.6586	3.81E-02
ENSMUSG00000029298	Gbp9	0.6249	1.93E-02
ENSMUSG00000029563	Foxp2	0.6198	1.91E-02
ENSMUSG00000028004	Npy2r	0.6131	1.22E-03
ENSMUSG00000035150	Eif2s3x	0.5979	6.90E-12
ENSMUSG00000000787	Ddx3x	0.5261	1.78E-11
ENSMUSG00000038859	Baiap2l1	0.4908	2.22E-02
ENSMUSG00000025332	Kdm5c	0.4857	3.81E-02
ENSMUSG00000046500	Fam19a4	0.4832	4.56E-02
ENSMUSG00000090570	Gm17041	0.4514	2.79E-02
ENSMUSG00000045657	Pcdhb10	0.4416	2.27E-03
ENSMUSG00000024079	Eif2ak2	0.4162	1.96E-02
ENSMUSG00000037613	Tnfrsf23	0.4111	2.79E-02
ENSMUSG00000087174	5530601H04Rik	0.4021	9.89E-05
ENSMUSG00000082286	Pisd-ps1	0.3990	6.41E-03
ENSMUSG00000051486	Pcdhb11	0.3969	1.93E-02
ENSMUSG00000032578	Cish	0.3930	3.39E-03
ENSMUSG00000045498	Pcdhb3	0.3913	5.38E-03
ENSMUSG00000023795	Pisd-ps2	0.3900	1.60E-05
ENSMUSG00000031226	Pbdc1	0.3779	9.33E-09
ENSMUSG00000029086	Prom1	0.3751	9.82E-04
ENSMUSG00000027765	P2ry1	0.3494	2.74E-03
ENSMUSG00000092223	Gm19807	0.3472	2.22E-02
ENSMUSG00000042686	Jph1	0.3471	7.33E-03
ENSMUSG00000044338	Aplnr	0.3426	4.59E-02
ENSMUSG00000030235	Slco1c1	0.3215	4.03E-04
ENSMUSG00000034738	Nostrin	0.3068	2.35E-02
ENSMUSG00000097571	Jpx	0.2993	3.51E-02
ENSMUSG00000031480	Thsd1	0.2658	1.67E-02
ENSMUSG00000025586	Cpeb1	0.2627	2.57E-02
ENSMUSG00000031673	Cdh11	0.2572	6.31E-03
ENSMUSG00000029802	Abcg2	0.2447	2.42E-02
ENSMUSG00000019647	Sema6a	0.2253	2.51E-02
ENSMUSG00000087528	9830144P21Rik	0.2246	1.51E-02
ENSMUSG00000033308	Dpyd	0.2231	6.62E-03
ENSMUSG00000049493	Pls1	0.2106	2.22E-02
ENSMUSG00000022321	Cdh10	0.2071	5.73E-03
ENSMUSG00000037709	Fam13a	0.2069	4.47E-02
ENSMUSG00000027397	Slc20a1	0.1860	1.48E-02
ENSMUSG00000018733	Pex12	0.1784	4.75E-02
ENSMUSG00000037134	Prmt9	0.1748	2.85E-02
ENSMUSG00000024621	Csf1r	-0.1905	6.02E-03
ENSMUSG00000044197	Gpr146	-0.2027	9.82E-04
ENSMUSG00000061833	Gm6311	-0.2058	4.40E-02
ENSMUSG00000031828	Klhl36	-0.2291	4.78E-02
ENSMUSG00000026090	2010300C02Rik	-0.2318	4.75E-02
ENSMUSG00000051504	Siglech	-0.2514	3.14E-02
ENSMUSG00000027997	Casp6	-0.2687	1.19E-02
ENSMUSG00000024292	Cyp4f14	-0.2704	1.96E-02
ENSMUSG00000058715	Fcer1g	-0.2779	3.95E-02
ENSMUSG00000021643	Serf1	-0.2841	3.69E-02
ENSMUSG00000097680	Gm26642	-0.2894	3.39E-03
ENSMUSG00000117924		-0.2903	4.20E-02
ENSMUSG00000018008	Cyth4	-0.3091	1.92E-02
ENSMUSG00000044551	9930012K11Rik	-0.3095	3.81E-02
ENSMUSG00000024695	Zfp91	-0.3106	3.03E-02
ENSMUSG00000039410	Prdm16	-0.3158	4.23E-02
ENSMUSG00000020374	Rasgef1c	-0.3198	4.01E-03
ENSMUSG00000003283	Hck	-0.3263	3.81E-02
ENSMUSG00000052192	Gm5963	-0.3299	2.95E-02
ENSMUSG00000036905	C1qb	-0.3309	1.91E-02
ENSMUSG00000034796	Cpne7	-0.3508	2.22E-02
ENSMUSG00000005951	Shpk	-0.3569	7.98E-03

Gene ID	Gene name	log2(FC)	FDR
ENSMUSG00000027400	Pdyn	-0.3600	5.73E-03
ENSMUSG00000050248	Evc2	-0.3610	5.38E-03
ENSMUSG00000073424	Cyp4f15	-0.3648	3.14E-02
ENSMUSG00000086938	4930481A15Rik	-0.3715	5.73E-03
ENSMUSG00000031805	Jak3	-0.3740	3.03E-02
ENSMUSG00000079235	Ccdc13	-0.3829	6.91E-03
ENSMUSG00000001741	Il16	-0.4013	6.91E-03
ENSMUSG00000089544	Gm25837	-0.4064	4.78E-02
ENSMUSG00000023153	Tmem52	-0.4067	3.81E-02
ENSMUSG00000029597	Sds	-0.4106	1.38E-02
ENSMUSG00000026278	Bok	-0.4201	2.06E-03
ENSMUSG00000038732	Mboat1	-0.4316	2.75E-02
ENSMUSG00000023067	Cdkn1a	-0.4439	3.95E-02
ENSMUSG00000026480	Ncf2	-0.4583	2.42E-02
ENSMUSG00000034842	Art3	-0.4790	2.28E-03
ENSMUSG00000005373	Mlxipl	-0.4907	3.81E-02
ENSMUSG00000001918	Slc1a5	-0.5009	3.81E-02
ENSMUSG00000051212	Gpr183	-0.5009	1.19E-02
ENSMUSG00000022715	Tmem114	-0.5046	3.11E-02
ENSMUSG00000082433	Gm9025	-0.5113	2.30E-04
ENSMUSG00000114429	AC113060.1	-0.5113	2.30E-04
ENSMUSG00000027315	Spint1	-0.5421	5.38E-03
ENSMUSG00000030711	Sult1a1	-0.5489	6.32E-04
ENSMUSG00000040428	Plekha4	-0.5517	2.42E-02
ENSMUSG00000071713	Csf2rb	-0.5602	2.71E-02
ENSMUSG00000048794	Cfap100	-0.5742	3.90E-03
ENSMUSG00000087371	Gm15541	-0.5835	1.19E-02
ENSMUSG00000116772		-0.5882	3.81E-02
ENSMUSG00000053198	Prx	-0.5964	1.19E-02
ENSMUSG00000092085	Gm17224	-0.6013	2.87E-02
ENSMUSG00000007122	Casq1	-0.6037	4.02E-05
ENSMUSG00000021676	Iqgap2	-0.6047	4.05E-02
ENSMUSG00000045573	Penk	-0.6260	1.83E-08
ENSMUSG00000032355	Mlip	-0.6338	1.67E-02
ENSMUSG00000086165	Gm15690	-0.6355	4.93E-02
ENSMUSG00000002104	Rapsn	-0.6411	1.68E-03
ENSMUSG00000085996	A830012C17Rik	-0.6476	2.77E-12
ENSMUSG00000043924	Ncmap	-0.6633	2.19E-05
ENSMUSG00000066058	Cldn19	-0.6986	1.82E-02
ENSMUSG00000024227	Pdzph1	-0.7079	7.89E-05
ENSMUSG00000087589	D430040D24Rik	-0.7103	2.77E-03
ENSMUSG00000067219	Nipal1	-0.7344	3.07E-03
ENSMUSG00000059900	Tmem40	-0.7579	9.31E-03
ENSMUSG00000006457	Actn3	-0.7755	3.81E-02
ENSMUSG00000016942	Tmprss6	-0.7824	8.40E-03
ENSMUSG00000046337	Fam178b	-0.7841	2.72E-04
ENSMUSG00000028475	Spaar	-0.7990	6.74E-04
ENSMUSG00000046922	Gpr6	-0.7994	3.79E-04
ENSMUSG00000012428	Steap4	-0.8071	1.86E-02
ENSMUSG00000105518	Gm42674	-0.8426	3.11E-02
ENSMUSG00000001020	S100a4	-0.8532	3.51E-02
ENSMUSG00000079415	Cntf	-0.8533	4.08E-05
ENSMUSG00000038086	Hspb2	-0.8741	2.19E-05
ENSMUSG00000071714	Csf2rb2	-0.8747	3.35E-02
ENSMUSG00000002289	Angptl4	-0.8857	1.83E-07
ENSMUSG00000104379	Gm37509	-0.9025	1.14E-05
ENSMUSG00000024517	Grp	-0.9127	4.09E-06
ENSMUSG00000032719	Sbspon	-0.9475	3.08E-07
ENSMUSG00000038541	Srd5a2	-0.9573	2.23E-02
ENSMUSG00000005220	Corin	-0.9781	1.81E-02
ENSMUSG00000073535	Gm5532	-1.0461	1.70E-02
ENSMUSG00000118365		-1.1792	4.01E-03
ENSMUSG00000102717	Gm37759	-1.2265	4.20E-02
ENSMUSG00000019278	Dpep1	-1.2952	6.20E-06
ENSMUSG00000009350	Mpo	-1.3646	2.30E-04
ENSMUSG00000022651	Retnlg	-1.3934	5.73E-03
ENSMUSG00000025480	Syce1	-1.5687	4.33E-04
ENSMUSG00000078365	Mos	-1.7320	1.44E-02
ENSMUSG00000106729	Gm43165	-1.8775	4.11E-03
ENSMUSG00000070368	Prok1	-1.9052	1.29E-03
ENSMUSG00000026822	Lcn2	-1.9636	4.28E-08
ENSMUSG00000064057	Scgb3a1	-2.1625	5.38E-03
ENSMUSG00000094613	A630076J17Rik	-2.1876	1.49E-04
ENSMUSG00000101059	Gm4017	-2.2965	1.72E-06
ENSMUSG00000050359	Sprr1a	-3.1135	2.10E-02
ENSMUSG00000056673	Kdm5d	-10.9214	1.34E-126
ENSMUSG00000069049	Eif2s3y	-11.5334	2.22E-94
ENSMUSG00000068457	Uty	-11.5861	7.61E-84
ENSMUSG00000069045	Ddx3y	-11.6740	9.46E-79

Table S2: IPA analysis of male-female DEG/Regulator effect

From Molecule(s)	Relationship Type	To Molecule(s)
CSF1R	causation	Recruitment of leukocytes
CSF1R	causation	Recruitment of myeloid cells
CSF2RB	causation	Recruitment of leukocytes
CSF2RB	causation	Recruitment of myeloid cells
HCK	causation	Recruitment of leukocytes
HCK	causation	Recruitment of myeloid cells
IL16	causation	Recruitment of leukocytes
IL17A	causation	Recruitment of leukocytes
IL17A	causation	Recruitment of myeloid cells
IL17A	expression	IL16
IL17A	expression	IL17A
IL17A	expression	LCN2
IL17A	expression	MPO
IL17A	protein-protein interactions	IL17A
IL17A	transcription	LCN2
Immunoglobulin	causation	Recruitment of leukocytes
Immunoglobulin	causation	Recruitment of myeloid cells
Immunoglobulin	expression	CSF1R
Immunoglobulin	expression	CSF2RB
Immunoglobulin	expression	HCK
Immunoglobulin	expression	IL17A
Immunoglobulin	expression	Immunoglobulin
Immunoglobulin	expression	LCN2
Immunoglobulin	expression	MPO
Immunoglobulin	localization	IL17A
Immunoglobulin	regulation of binding	IL17A
Immunoglobulin	regulation of binding	Immunoglobulin
Immunoglobulin	transcription	IL17A
LCN2	causation	Recruitment of leukocytes
LCN2	causation	Recruitment of myeloid cells
MPO	causation	Recruitment of leukocytes
MPO	causation	Recruitment of myeloid cells

Interpretation of male and female DEG with IPA software, using the Regulator Effects algorithm. The Regulator Effects algorithm connects upstream regulators, dataset molecules and downstream functions or diseases affected in the dataset to generate a hypothesis that can explain how the activation or inhibition of an upstream regulator affects the downstream target molecule expression and the impact of the molecular expression on functions and diseases. The

algorithm goes through one or more iterations to merge upstream and downstream results from the Upstream Regulator. The networks are merged only if the overlap of targets has possible statistical significance (Fisher's Exact Test p-value of <0.05). For each network, a Consistency Score is calculated that rewards for paths from regulator->target->disease or function that are consistent. Higher scoring hypotheses are those with more consistent causal paths represented by a high Consistency Score.
